# Development of Emulsified Concentrate (EC) formulation of *Mentha piperita* oil for control of mosquito larvae

**DOI:** 10.1186/1475-2875-11-S1-P58

**Published:** 2012-10-15

**Authors:** Peevush Kumar, Sapna Mishra, Anushree Malik, Santosh Satya

**Affiliations:** 1 Applied Microbiology laboratory, Centre for Rural Development & Technology Indian Institute of Technology Delhi, New Delhi-110016, India

## Background

Malaria is a leading cause of morbidity and mortality with an estimated 2.2 billion reported cases and 0.7 million annual deaths [1]. Continued research for malaria control although has resulted in significant reduction in malaria mortality rate, investigative measures are needed to address the escalating cost and threat of pesticide resistance. In this direction, biological control agent such as essential oil presents a good alternative [2-4]. However, the commercial applicability of essential oils for mosquitoes control is plagued by their slow action, variable efficacy and lack of suitable/user friendly end product [5]. Thus, in the present study Emulsified concentrate (EC) formulation of *Mentha piperita* oil, an effective mosquito larvicide [2,3], was prepared and characterized.

## Methods

Emulsified concentrate (EC) formulation of *M. piperita* oil was prepared by mixing (w/w) 40% oil, 45% aeromax, 3% butanol-1 and 12% surfactants (CABS-70 and NP-20), which was homogenized at 5000 rpm for 30 min. prepared EC formulation was characterized for its creaming kinetics, droplet size distribution, zeta potential, viscosity, pH, conductivity and flash point. Formulation was also assessed for the effect of different temperature (4, 15, 30, 45 and 60°C) under storage condition.

## Results

EC formulation was reddish in appearance with a creaming volume of 0.4% and particle size of 563.7 nm. Zeta potential of the resultant emulsion was -45.9 mV. The EC formulation showed a viscosity of 25 cP while its pH was slightly acidic at 6.6. The value of conductivity and flash point for EC was 8.8 mS/cm and 92°C, respectively. EC formulation was stable at the temperature of 4, 15, 30°C and to some extent even at 45°C. The temperature of 60°C was not suitable for EC stability and resultant emulsion showed phase separation.

## Conclusions

Low creaming volume and negative value of Zeta potential indicated a stable emulsion. Relatively high flash point of the product observed in this study, makes it less volatile and safer to transport and handle. Temperature stability for storage signifies formulation utility in different conditions of thermal variation. The prepared formulation being applicable as emulsion could be suitably used for control of mosquitos’ larvae in various aquatic habitats.
